# Healthcare providers’ perspectives on a mobile app for patients with type 2 diabetes: A focus group study to enhance user-centered digital health design

**DOI:** 10.1177/20552076261435084

**Published:** 2026-05-16

**Authors:** Andreia Pinto, Glória Conceição, Emília Moreira, Paulo Santos, Alberto Freitas

**Affiliations:** 1 RISE-HEALTH, Faculdade de Medicina, 26706 Universidade do Porto, Porto, Portugal; 2 Departamento de Medicina da Comunidade, Informação e Decisão em Saúde (MEDCIDS), Faculdade de Medicina, Universidade do Porto, Porto, Portugal

**Keywords:** Focus group, type 2 diabetes mellitus, mobile apps, healthcare providers, self-management, behavior change

## Abstract

**Introduction:**

Technologies to help patients with type 2 diabetes mellitus (T2DM) have increased and have shown promising results in supporting self-management. However, not all applications consider healthcare providers’ knowledge in designing and developing these tools, which may impact adoption and effectiveness. This study examined healthcare providers’ perspectives on how mobile phone technologies can support patients with T2DM in self-managing their condition and inform user-centered design.

**Methods:**

We conducted two online focus group sessions with nine healthcare providers involved in T2DM care. A semistructured guide was used to explore the participants’ perspectives on adopting mobile apps. Transcribed narratives were transcribed verbatim and analyzed using thematic analysis. The report followed COREQ guidelines for qualitative studies.

**Results:**

Five main themes emerged: (1) diabetes—challenges and self-care; (2) the use of technologies in managing T2DM; (3) the market for diabetes management tools; (4) suggestions for ideal app features; and (5) the role of healthcare providers. Healthcare providers acknowledged the benefits of mobile apps in enhancing patient engagement to manage type 2 diabetes. Participants also pointed out the barriers to full implementation, such as usability challenges, patient digital literacy, and integration with clinical workflows. They also considered that patients with T2DM should provide feedback on digital health designs.

**Conclusion:**

Healthcare providers’ involvement in developing an app ensures alignment with clinical practices and patients’ needs. The study's findings support the user-centered design of digital tools tailored to managing T2DM and may inform future digital health design and evaluation for T2DM or other chronic conditions.

## Introduction

Type 2 diabetes mellitus (T2DM) is a chronic disease with a high prevalence worldwide, affecting around 10.5% of the adult population. The International Diabetes Federation estimates that by 2030, 643 million people will live with diabetes.^1^ This high prevalence significantly burdens health systems due to high direct treatment expenditures and complications management.^2,3^

T2DM has a genetic component, but lifestyle choices significantly influence its development. Poor eating habits, lack of physical activity, and being overweight are key behavioral factors that contribute to the progression of the disease and the onset of complications.^4,5^

Good glycaemic control is crucial in these patients.^6^ Healthcare providers are essential in developing disease management strategies, considering patient-centeredness and motivational factors.^7^ Patients’ self-care is also crucial for disease management and patient wellbeing.^8^ Self-management is complex as it involves knowledge about medications, diet, physical exercise, and the challenges of developing attitudes and skills for true health gains.^9^ However, there are many barriers to an effective self-management of T2DM, such as the lack of motivation, low health literacy, limited economic possibilities, and lack of time and communication with healthcare providers accompanying the patient.^10^ Following a healthy diet and promoting weight loss are crucial, but they are not always easy. Diet is a complex phenomenon influenced by behavioral, cultural, and social determinants. The better option for the patient may be the one that they can maintain, given their own preferences, culture, social context, and time.^11^ This favors adherence.^12^ Additionally, health literacy also plays a pivotal role, improving the patients’ ability to make informed and healthier decisions. Inadequate health literacy in patients with T2DM is quite common. Patients often struggle to manage their disease through nutrition. This has an important negative impact on health, being associated with worse HbA1c and lipid profiles.^13^

Technologies for managing chronic conditions using mobile phone applications have become an increasingly popular tools that positively influences clinical outcomes.^14,15^ Nevertheless, these technologies have a high dropout rate after just a few months. Attrition rates found in a meta-analysis of app-based interventions for chronic diseases were 40%, ranging between 9% and 82% in randomized controlled trials (RCT) and 49% (25–84%) in observational studies.^16^ One possible explanation is that many apps did not incorporate feedback from healthcare providers and patients during their design process.^17^ As a result, technology may be abandoned by those who would benefit most from its use and continuous improvement. User-centered design, as defined by ISO 9241-210, is an iterative process that actively involves users in the development of a system, ensuring it aligns with their needs, characteristics, and the context in which it will be used.^18^ Their implementation is associated with higher usability, satisfaction, and fewer post-implementation concerns.^19^ However, the healthcare professionals may act as a proxy for user-centered design, as they are aware of these patients’ medical needs, and their insights may be quite relevant to developing this application type. Their extensive experience in managing patients with T2DM provides them with a valuable understanding of these patients’ needs and how this type of tool can benefit their daily condition management.^9^ Moreover, they can identify self-management barriers, as they often detect these difficulties during appointments. Previous studies have shown the benefits of incorporating healthcare providers’ opinions in developing mobile applications. In a study evaluating primary care professionals’ perspectives on a text messaging intervention, the authors emphasized that involving them is crucial, as they understand the needs of patients with T2DM.^20^ Their feedback can help increase patient engagement with the technology and thus make the most of its potential, which is essential for behavior change.^21^ However, to create mobile apps that have a user-centered design, there is a scarcity of studies that evaluate the perceptions of healthcare providers considering the utility of mHealth and its features to improve self-management and quality of care in patients with diabetes.^3^ The Food Friend project (https://itea4.org/project/food-friend.html) aims to develop a toolset that can measure the food intake of patients with T2DM in a way that requires minimal patient input and turns it into personalized feedback.

The focus group methodology is a well-established data collection strategy that promotes discussion of a topic of interest to all participants, thereby gathering diverse perspectives on a given issue.^22^

The present study aimed to explore healthcare providers’ knowledge and perspectives on using mobile phone technologies to assist patients with T2DM in self-managing their condition and inform a design based on the patients’ needs. Additionally, we sought to determine the healthcare providers’ opinions on the mobile phone application being developed for the Food Friend project and identify the most effective strategies for its implementation.

## Methods

### Study design

We conducted a qualitative study using the focus group methodology. Two semistructured online focus groups explored healthcare providers’ perspectives on technologies, particularly a mobile app, to improve behavior change and self-management in patients with T2DM. The focus group methodology was chosen because it encourages participant interactions and generates new ideas, such as reflections and debates.^22^ Consolidated Criteria for Reporting Research (COREQ) guidelines were followed to report research data (Supplemental File).^23^

### Participants

A purposive sample was used to recruit the participants. Our inclusion criteria were healthcare providers who work directly with T2DM, in particular, general practitioners, endocrinologists, nurses, and dietitians, ensuring a good knowledge of the condition across various areas of diabetes care. Healthcare providers were invited to participate by email, which explained the project, its aim, and the study's methodology through contacts provided by the team's researchers. This allowed us to send 12 emails to healthcare providers, 10 of whom agreed to participate. This email also contained a link that healthcare providers had to complete if they were interested in participating in the study. Participants were asked to fill out a short questionnaire that included some sociodemographic characteristics, and all gave written informed consent.

Before the consensual date, we shared a manual presenting the mobile application developed within the project context and videos illustrating how it works (showing all the features) to be viewed before the virtual focus group session. About 3 days before the session, a new reminder email was sent with the link and password to access the online session.

### Data collection

A semistructured focus group interview guide was developed by the authors AP (MSc), GC (PhD), and PS (PhD) according to the study objectives and based on the literature (Table 1).^24,25^ The guide was not piloted; it was iteratively refined by the multidisciplinary author team based on the literature. Their professional backgrounds are in nutrition, cardiovascular health, and general and family medicine. PS moderated the sessions, and he is a general practitioner with expertise in conducting qualitative research methods and the director of the telemedicine course at the university. Two facilitators took field notes during the sessions.

**Table 1.** Focus group semistructured interview guide.

	
1. Use of digital technologies in people with T2DM	What are the advantages and difficulties of using new technologies for people with T2DM?
	How do you use these technologies in your clinical practice?
	What can you do to convince patients to change their lifestyles?
2. Food Friend app	Can it empower patients to manage their behavior?
	Is this app great?
	Do the patients use this kind of technology in their daily lives?
	What does this application lack in optimizing its goal?
	How can it help to facilitate communication with patients?
3. Without technological constraints, where could technology aid patients with T2DM	Without technological constraints, where would we use technology to aid patients with T2DM?
	Considering those at the beginning of the disease diagnosis, where are lifestyle changes significant? What do we need and do not have in this mobile application?

T2DM: type 2 diabetes mellitus.

The session started with a general introduction to the study, an explanation of how the focus group would work, and the main topics to discuss. In each session, we tried to have each healthcare provider class representative enrich the discussion. However, one participant dropped out at the beginning of the focus group session due to a personal problem. Thus, five healthcare providers attended the first session, which took place in May 2023, and four participated in the second session, which occurred in June 2023. The focus group sessions ranged from 75 to 90 minutes, respectively. The sessions were conducted through the online video conferencing software Zoom^©^ and were visually and audio recorded using this software. As the team had visual and audio recordings and also notes of the sessions to check accuracy, the transcripts were not made available to the participants. There were no repeated interviews. The Ethics Committee of the Faculty approved the study.

### Data analysis

The first author transcribed the focus group sessions verbatim into a Word document. After transcription, the recordings were deleted, and the focus group participants’ quotes were anonymized.

The transcripts were analyzed using a thematic content analysis, in which information was grouped into themes, categories, and subcategories. The content was analyzed, deriving information from the data (inductive approach), using the following steps: (1) Initially, the entire document was read to familiarize the researchers with the text; (2) the information was color-coded to make it easier to identify similar codes and the differences between them; (3) similar codes were identified and grouped to create main themes. Based on identifying the main themes, the categories and subcategories were then organized and labeled with clear and representative titles; and (4) lastly, the results were interpreted. Two coders were involved. The first author performed the initial data analysis, and afterward, together with the second author, they checked all quotations and agreed on all themes generated, guaranteeing their reliability. Coding disagreements were resolved by discussion to consensus; no third reviewer was required. After analyzing the two focus group sessions, we found that no additional information emerged between the first and second sessions, indicating that data saturation may have been reached.

Descriptive statistics were presented as the median and range for quantitative variables and the frequencies and respective percentages for qualitative variables. The qualitative data covering the focus group sessions were organized and analyzed manually, with no software being used.

## Results

Participants’ characteristics are presented in Table 2. Nine healthcare providers (three general practitioners, two endocrinologists, two nurses, and two dietitians) participated in the sessions. Most participants were female (55.6%), and the median age was 41. The professionals’ clinical practice in managing T2DM was wide-ranging, with professionals having less than 5 years of experience (22.2%) and very experienced professionals with more than 20 years of experience (33.3%). Most of them worked in the public sector (77.8%). Healthcare providers were divided into two groups, the first containing five participants and the second containing four participants.

**Table 2.** Characteristics of the focus groups’ participants (n = 9).

Characteristics	n = 9
Gender, n (%)	
Female	5 (55.6)
Male	4 (44.4)
Age, median (range)	41.0 (27-66)
Profession, n (%)	
General practitioner (GP)	3 (33.3)
Endocrinologist	2 (22.2)
Nurse	2 (22.2)
Dietitian	2 (22.2)
Experience in T2DM (years), n (%)	
Less than five years	2 (22.2)
5–10 years	1 (11.1)
11–15 years	2 (22.2)
16–20 years	1 (11.1)
More than 20 years	3 (33.3)
System of health, n (%)	
Public	7 (77.8)
Private	1 (11.1)
Both	1 (11.1)

T2DM: type 2 diabetes mellitus.

Focus group thematic analysis identified five themes (Table 3): (1) diabetes—challenges and self-care; (2) the use of technologies in managing T2DM; (3) the market for diabetes management tools; (4) suggestions for ideal app features; and (5) the role of healthcare providers. In total, 14 categories and 25 subcategories were also identified. The first three categories provided more general information regarding the target population and their technological use and the last two (themes 4 and 5) directly addressed our main aim, with insights considering the app's features and the role of healthcare providers in this kind of mobile application.

**Table 3.** Themes, categories, and subcategories identified in the focus group sessions.

Themes	Categories	Subcategories
**1.** **Diabetes: challenges and self-care**	Diabetic patients’ characteristics	-
	Differences in motivation and use of technology in T1DM and T2DM	-
	The main difficulties in diabetes management	-
**2.** **The use of technologies in managing T2DM**	Target group	-
	Challenges and barriers	Digital literacy and age
		Misinformation/Health literacy
		Ingrained eating habits
		Family support
		Duration of app use
		Meal registration
	Advantages	Assistance in clinical practice
		Gain of knowledge
		Integration into people's routines
**3.** **The market for diabetes management tools**	Marketing	
	Need for app assessment by people with T2DM	-
		-
**4.** **Suggestions for ideal app features**	Features	Gaming and setting goals
		Coaching/Feedback
		Physical activity
		Integration with other apps
		Family support
		Diet
		Schedule of appointments/exams
	Customized app	Behavior change
		Complex patients
		Managing expectations
		Simplicity
	Artificial intelligence	Automation
	Education	Empowerment
		Health literacy
		Labeling
**5.** **The role of healthcare providers**	Communication	-
	Data monitoring	Data interpretation

T2DM: type 2 diabetes mellitus.

A selected number of direct quotes from the participants were used to illustrate their experiences (Table 4). The number of participants was placed in front of each quote to demonstrate who pronounced each sentence.

**Table 4.** Quotes illustrating the themes and categories generated by the group interaction.

**Theme**	**Categories**	**Quotes**
**Diabetes: challenges and self-care**	Diabetes patients’ characteristics	“Our problem is that type 2 diabetes in Portugal is mainly among people with lower income, less literacy, and years of education, and who have smartphones and apps, but it is mainly for social purposes.” (P6)
	Differences in motivation and use of technology in T1DM and T2DM	“In type 2 diabetes, few people use applications. In type 1 diabetes, it is more common.” (P1) “The big question among type 1 and type 2 diabetic patients is that type 1 diabetic patients are much more motivated, much more sensitive to the use of this type of technology.” (P9)
	The main difficulties in diabetes management	“Where I see patients with diabetes having the most difficulty is realizing which foods they can’t eat, or eat sporadically. The diet issue is very complicated.” (P2) “Patients’ perception that diet and physical activity are also part of the treatment.” (P3) “In diabetes type 2, we see several patients who are often unaware of the importance of maintaining care over time and being aware of these controls.” (P8)
**The use of technologies in managing T2DM**	Target group	“I think that it is at the beginning of the disease that we can eventually direct the app and excuse the term ‘selling’ the app.” (P9) “I was wondering which target group (…) currently between 40 and 55 years old would be. So, they have a recently diagnosed diabetes and are sufficiently motivated to use this type of technology.” (P7)
	Challenges and barriers	“This is where the big challenge lies: presenting an application that they feel will be useful to them.” (P9) “That the app is useful in the long term.” (P2) “The age gap is notorious (…) this type of technology is beneficial, yes, but perhaps at this moment there is a population range that still cannot fully benefit from it.” (P3) “I can tell you that less than 10% of my patient list uses technology. Thus, they have a very low digital literacy.” (P5) “Another thing that I think this app has to try to work on a bit is fighting the misinformation that exists in this area, and that's very difficult.” (P2) “Many patients have no idea how they came to have type 2 diabetes, what happened to lead them to have it.” (P8) “One of the deepest barriers is our health systems, which are closed to this type of application.” (P2) “The phenomenon of apps that are used for a while and then fade away has already been studied, except for social apps.” (P6) “It makes sense to regulate diet. I believe that it is possible to do a good job with this approach, but it is obvious that we will not have well-measured quantities; that is impossible.” (P5) “I don’t see how photography can cover so much diversification.” (P4)
	Advantages	“It's a great strategy for combining these three things into one (diet, exercise, and medication), which is what I haven't seen so far, at least in the applications I know.” (P5) “These applications are very important for health literacy.” (P8) “It's also good for people to understand what they are eating and the quantities so that they can get some kind of feedback.” (P8) “It allows them to have a record on a device that they use daily and that integrates their lives.” (P3) “I think the app could be an extra form of support that the health professional is unable to provide.” (P4)
**The market for diabetes** **management tools**	Marketing	“So, what's the best way to sell a product like this? It is important to see the problems with the comprehensibility of the text and if the way it's put together is suitable for selling what needs to be sold. (…) To improve the app, put it to several people and ask, “What do you think? Does this sell or not? Understandable or not? Would you accept this or not? What are the things that people see in it?” (P6)
	Need for app assessment by people with T2DM	“Involving people with diabetes can be useful because they will say what they would like to see in the app.” (P1)
**Suggestions for ideal app features**	Features	“Gaming and achieving goals can be a way for people to stay connected and maintain continued use over time.” (P3) “This notion of feedback and the people getting feedback on the results of what is happening gives people with diabetes much security.” (P2) “But it could also be associated with the prescription of physical exercise, which has to take into account the person's clinical characteristics.” (P7) “It could be useful to increase the impact of the application's use by collecting information from third-party devices.” (P7) “If a person could create a list of commonly used foods themselves, there could potentially be a basis for easily identifying the weight of these foods.” (P1) “I'd like to bring to discussion the issue of drinks, particularly alcoholic drinks, the quantification of calories, and the impact in terms of the glycemic index.” (P3) “It might be interesting to include in the application the possibility of having a calendar to add dates, for example, of appointments or analyses. It could also give reminders, for example, about water intake.” (P3) “I'm still not very clear about the time it takes to use it, the availability that using this application requires.” (P9)
	Customized app	“But do not forget that many of these patients are also complex patients. At a more advanced stage in the development of the mobile application, this should be one of the precautions to be considered.” (P3) “It has to be very simple; the patient has to do very little for it to work, and the essential steps should be all very automated.” (P2)
	Artificial intelligence	“I think that anything that the app asks us for our opinion should be optional; for example, there's a photo of bread, I think it should automatically suggest what it is; only if the person wants to change it, he/she should go there and change.” (P2) “I speak for myself, if I had to insert it by hand, I do not know if I would insert anything at all.” (P7)
	Education	“Health education is an essential step, especially in type 2 diabetes, where the mechanism of the disease is largely due to the person's habits.” (P8) “Educating patients is essential, teaching them what carbohydrates are. People think that diabetes comes from sugar; they do not know that bread, pasta, and rice are contributing to their getting out of control of the disease.” (P8)
**The role of healthcare providers**	Communication	“It was important that through the app, they had some way of connecting with us, the family nurse, the family doctor, but with limits! (…)” (P5) “I don't have such a concrete vision or idea (…) because otherwise we, healthcare providers, are going to be slaves to these apps.” (P1) “The question of whether the app can be a good tool for us professionals, I don't think that's the point. I think it has to be a good tool for the patient.” (P5)
	Data monitoring	“If we have a continuous record of people's habits, whether in terms of diet or physical exercise, I hope we will also be able to provide more personalized advice (…)” (P3) “Another thing I was going to suggest is sharing data, which I think is very valuable.” (P2) “What fascinates me about this application is that it monitors things that we are not monitoring because I have realized that we control better what we measure.” (P2)

T2DM: type 2 diabetes mellitus.

### Diabetes: Challenges and self-care

Healthcare providers discussed various T2DM-related issues, providing an understanding of the characteristics of people living with diabetes to contextualize the problem and thus as a starting point for discussing technology in this population. Therefore, three distinct categories emerged in this theme.

#### Characteristics of patients with diabetes

The key sociodemographic and economic characteristics of individuals with diabetes were examined, particularly how these factors can negatively impact the progression of the disease. It was highlighted that these challenges can complicate both disease management and the use of technologies.

#### Differences in motivation and use of technology in type 1 diabetes mellitus (T1DM) and T2DM

Healthcare providers considered the two main types of diabetes to be very different in motivation. Patients with T2DM were pointed out as having lower motivation and propensity to use technology compared to patients with T1DM.

#### The main difficulties in diabetes management

Healthcare providers have varying opinions on the main challenges faced by patients with diabetes in managing their disease. However, most of them agree that patients lack knowledge and face difficulties in controlling T2DM when it comes to their diet.

### The use of technologies in managing T2DM

The technologies currently used to control T2DM and the technological tools that could positively impact the management of the disease were discussed. In this way, the group addressed three main categories and nine subcategories about this topic.

#### Target group

All the focus group session participants agreed that the target audience would benefit from technologies in managing type 2 diabetes. In particular, mobile phone applications would be helpful for people with a recent diagnosis of T2DM due to their degree of motivation and ability to improve the progression of the disease.

#### Challenges and barriers

The healthcare providers highlighted several challenges in patients with T2DM's use of technology. One of the main points that drew attention was the development of an application that patients feel will be useful to them, especially in the long term.

At the individual level, healthcare providers emphasized age, lack of digital and health literacy, and widespread misinformation as barriers to frequently using this type of technology, particularly mobile phone applications. At the health system level, the participants pointed out that the existence of a health system closed to external applications is challenging, so they cannot transfer the data from the applications to clinical practice. The healthcare providers also emphasized that they considered the phenomenon of apps lasting longer challenging. As a result, they reported that there must be a way to keep people interested in using the app so that they can see an advantage in using it. Moreover, most participants reported that continuously recording meals can be challenging, as it is difficult to have absolute control over what is eaten, and even through photography, this process can be inaccurate.

#### Advantages

Several advantages have been listed regarding the use of T2DM technologies. The fact that the population is becoming more used to technology, in the opinion of healthcare providers, is a positive point for it to be used frequently and thus contribute to achieving the desired results. In addition, the fact that mobile phone applications can combine several components can make it easier to visualize the disease as a whole.

Notably, the participants recognized that the technologies could help patients with T2DM gain knowledge about their condition and be integrated into patients’ routines. Finally, it was identified that the use of technology could be seen as an aid in the clinical practice of healthcare providers, as it allows patients to reflect on the outcomes of their choices. Some professionals believed that this technology could act as extra care for patients.

### The market for diabetes management tools

The issue of commercializing this type of technology was also debated in the focus group sessions, being a relevant issue to try to persuade the target audience of the importance of its use.

#### Marketing

The participants expressed the need for a good marketing strategy behind the sale of apps.

#### Need for app assessment by people with T2DM

Besides that, there must be an evaluation of the technology by patients, as they could indicate what they would like to have in an application of this kind, reflecting their needs.

To optimize its usefulness on the market, the app must first be evaluated by patients with TD2M.

### Suggestions for ideal app features

Although all the participants considered mobile phone apps valuable tools to help manage T2DM, they made several suggestions when asked what the app evaluated in the context of the project could have improved or lacked technology. Four categories emerged from this theme, relating to the functionalities the app could have or would be helpful to improve, the importance of a personalized app, and the role that artificial intelligence and education can play in developing technologies.

#### Features

The participants highlighted the functionalities they felt would be fundamental for an app to help patients with T2DM effectively. Although the mobile application that the participants had the opportunity to evaluate in the context of this study contained some of the functionalities that had been suggested, some suggestions were given to improve the existing functionalities. The features highlighted were as follows: a gaming feature that at the same time gave patients a chance to create goals to see their progress; integration of participants into a community where they could share ideas, concerns, and achievements; a coaching/feedback feature based on the data entered into the app; the possibility of adding more types of exercise other than walking and the prescription of exercise by healthcare providers in the app; a calendar that allows people to schedule the appointments and exams; reminders for entering metrics into the app.

Several suggestions for improving the food input component were discussed, namely creating a list of commonly consumed foods, paying attention to drinks, and finally, the possibility of integrating the app with other applications to automate the data transfer process. Generally, the healthcare providers recognized that the app being developed has the potential to help people with diabetes. However, some were more reticent about the time patients will have to spend using it.

#### Customized app

Participants were a little reluctant regarding the use of an app, mainly when there was a need to introduce several health metrics. So, if it is not user-friendly, it could burden patients. The healthcare providers pointed out that the app would have to be simple and personalized to the needs of each participant, facilitating adherence to the app and, thus, behavioral change. One of the professionals also recalled that complex patients should be considered when customizing the app, as patients with T2DM often have other conditions.

#### Artificial intelligence

To simplify and encourage the use of the mobile application, it was noted that artificial intelligence could be used to automate the app's features and enable it to learn from mistakes.

#### Education

Education is one of the main requirements for the successful management of T2DM because even if patients have the tools, they may need to learn how to use them effectively. This pathology has a strong behavioral component, so patients’ choices significantly impact its progression. Healthcare providers raised concerns in this regard. They consider that an app should help manage the disease and, at the same time, improve health literacy.

### The role of healthcare providers

This topic addresses how this type of technology can benefit healthcare providers, and it was the one on which participants’ opinions differed.

#### Communication

Integrating applications into appointments and sharing data with healthcare providers was highlighted as attractive. A few participants pointed out that it could be even more interesting if the app could connect directly with the healthcare providers who follow the patient, which raised concerns from other colleagues because, in that case, the healthcare providers would be burdened with additional work.

#### Data monitoring

This feature type was considered very interesting once it gathers information that healthcare providers often do not collect in clinical practice. The participants considered that they could better control what was measured, and if they could measure more metrics, personalized advice could be more easily provided.

## Discussion

To the best of our knowledge, there is a lack of studies that explore healthcare providers’ perspectives regarding the use of technologies in managing T2DM in Portugal. Our findings reveal that healthcare providers view mobile applications as promising tools to enhance T2DM self-management, particularly by supporting patient engagement, improving understanding of daily behaviors, and complementing clinical care. At the same time, they identified important barriers to effective and sustained use, including low digital and health literacy, limited confidence in technology among older or socioeconomically vulnerable patients, and the burden of continuous data entry. Providers also emphasized systemic challenges, most notably the lack of integration with existing health information systems and concerns about long-term adherence, which they believe must be addressed for such tools to produce meaningful and lasting benefits. Together, these insights highlight the need for user-centered, workflow-aligned, and literacy-sensitive design strategies when developing digital health solutions for people living with T2DM.

Through thematic analysis of the focus group sessions, five main themes were identified: (1) diabetes—challenges and self-care; (2) the use of technologies in managing T2DM; (3) the market for diabetes management tools; (4) suggestions for ideal app features; and (5) the role of healthcare providers. These five themes covered 14 categories and 25 subsequent subcategories.

This study demonstrates that healthcare providers see mobile phone applications as valuable tools for managing T2DM and assisting patients with self-management, aligning with previous research.^3,26^ However, the focus group discussions revealed concerns about the functionality of these tools. To ensure effectiveness, careful planning and consideration of various issues are essential. Participants suggested potential improvements and features to enhance the effectiveness of these technologies.

Healthcare providers have observed that patients with T2DM often have sociodemographic and economic characteristics that may limit their ability or willingness to use technology. Several studies identified these patients as older people with lower literacy and education levels and fewer financial resources.^27,28^ The participants saw these conditions as factors that can hinder the management of T2DM and technology usage, as in a study conducted by Bashi *et al*.^29^ Additionally, patients with T2DM tend to show less motivation for management than those with T1DM, as the latter's symptoms worsen quickly when the disease is not well controlled.^30^

Healthcare providers agree that diet is a major challenge for patients with T2DM, complicating disease management. Patients need a clear understanding of their dietary choices. Previous studies have examined healthcare providers’ perspectives on nutrition-based mobile apps and found that they recognize these tools provide a clearer picture of food intake^31^ and many providers consider the apps to be superior to traditional methods for tracking patients’ dietary intake.^32^ It could increase patient awareness of nutritional needs, which could be a valuable tool if it has a user-friendly design.^31^ A systematic review also demonstrated that the use of a mobile health app can support the self-efficacy of the patients and improve their communication and the relationship with the provider in ambulatory and hospital settings.^33^ However, as our study found, they consider that integrating with other existing electronic systems should be easier.^34^

Therefore, our participants recommended that the app include educational resources to enhance knowledge about T2DM management, thus empowering them, a concept previously considered by different studies in this field.^3,20,35,36^ Participants also expressed some concerns about the app's utility over time. It should be a valuable tool that promotes long-term use for effective disease control. Some healthcare providers noted that not all patients with T2DM might have the necessary digital or health literacy.^20^ Thus, family support is crucial.^37^ Family should support relatives with diabetes regarding their meals and adopting a healthier diet, because otherwise, disease control could be compromised, even with the help of an app. Additionally, professionals stress that the app must be user-friendly^38^ and personalized to meet individual needs^35^ to foster positive behavioral change.^3^

Although not explicitly structured with any technology acceptance framework, the results of this study can be considered according to consolidated models on technological acceptance, namely the Technology Acceptance Model (TAM) and the Unified Theory of Acceptance and Use of Technology (UTAUT), which are useful for interpreting the dynamics involved in accepting the use of technology in the context of this pathology. The participants emphasized the utility and the ease of use of the mobile apps regarding the self-management of the disease by the patients with T2DM. It reflects the main constructs of TAM: “perceived usefulness,” which describes that the technology can enhance the performance in a particular task, in this case, in the control of the disease, and the “perceived ease of use,” which suggests that the technology should be easy to use. Regarding UTAUT, participants also pointed out that one of the barriers (i.e. lack of facilitating conditions) was the mechanisms for integrating this type of technology into health systems.

Regarding the features an app should include to manage T2DM effectively, healthcare providers emphasized the importance of several elements. These features should allow users to set goals,^38^ share information with a community,^36,39^ and provide feedback on the data introduced into the app, preferably in a positive and motivational reinforcement.^39^ Additionally, the app should have a calendar and reminders, along with the capability to connect to other applications.

The findings of this study reveal that healthcare providers have varying perceptions of the usefulness of technology in managing T2DM, primarily influenced by their familiarity with such tools. Moreover, while these tools are considered essential for integrating technology into clinical practice and offer measurements that cannot be constantly monitored, the most crucial aspect is the app's relevance to patients with T2DM. Many healthcare providers also expressed the importance of involving patients with T2DM in developing such an app, as they believe that patients are better equipped to articulate the features they would like to see. This aligns with recent studies^26,39^ that support this perspective.

### Strengths and limitations

This study presents both strengths and limitations. Opinions from diverse healthcare providers were gathered, including dietitians, nurses, and physicians, all experienced in working with patients with T2DM. Participants varied in professional experience, influencing their understanding of the disease and the use of technology in clinical practice.

The remote focus group sessions gained higher popularity during the COVID-19 pandemic and fostered effective discussions. This online option is cost- and time-saving, allowing for the recruitment of people who are usually very busy.^40^ As a result, we experienced high acceptance and low dropout rates in our study. It also contributes to reduce anxiety and logistical issues associated with in-person meetings.^41^ However, it may introduce the disadvantage of a lack of nonverbal cues (eye contact and body language), and people who lack digital literacy could be excluded.^42^ As participants feel more anonymous because they do not have to be physically present, some may drop out of the study more easily, which was not detected in this study. However, it is also this feeling of anonymity that often allows them to speak more freely about the topics in question.^43^ As suggested by Wettergren et al.,^44^ we conducted our online focus group sessions between three to five participants. In the literature, it is suggested that the quantity and quality of data obtained in online focus groups are comparable to those acquired in traditional focus groups.^41,43,45,46^ Regarding participants, we used a purposive sample, which may have introduced selection bias. Additionally, the study had a small nonrandom sample size, which limits the generalizability and transferability of the results. Despite this, Hennink et al.^47^ suggested that data quality is more critical than the number of participants in qualitative research. Nevertheless, a larger sample size could ensure better saturation data. Credibility was enhanced through analyst triangulation and an audit trail; however, we did not conduct member checking or formal reflexivity exercises. Most participants worked in urban areas, but some experienced treating patients from nonurban regions.

## Conclusion

Healthcare providers were optimistic about the benefits of using technology for self-managing T2DM, mainly through a mobile phone application. However, they still have concerns about the app's ease of use and the daily time commitment required to monitor the condition. The participants shared insights on essential features of the app, and integrating their insights ensures alignment with clinical practices and patient realities.

This study has helped refine these ideas, and the findings will guide the development of our intervention, which will certainly enrich our mobile app, particularly regarding the insights gained about the features. It may also contribute to future research on T2DM and other chronic conditions, serving as a basis for other mobile apps or focus group development. These considerations are essential for researchers, technology developers, and patients in creating effective health technologies.

## Supplemental Material

sj-pdf-1-dhj-10.1177_20552076261435084 - Supplemental material for Healthcare providers’ perspectives on a mobile app for patients with type 2 diabetes: A focus group study to enhance user-centered digital health design Supplemental material, sj-pdf-1-dhj-10.1177_20552076261435084 for Healthcare providers’ perspectives on a mobile app for patients with type 2 diabetes: A focus group study to enhance user-centered digital health design by Andreia Pinto, Glória Conceição, Emília Moreira, Paulo Santos and Alberto Freitas in DIGITAL HEALTH
